# From theory to bench confirmation or from bench to theory

**DOI:** 10.1186/s40635-016-0113-2

**Published:** 2016-12-09

**Authors:** Julien Demiselle, Frauke Tillmans, Andreas Koch, Peter Radermacher, Pierre Asfar

**Affiliations:** 1 Département de Réanimation Médicale et de Médecine Hyperbare, Centre Hospitalier Universitaire, Angers, Cedex 9 49933 France; 2 German Naval Medical Institute, Section for Maritime Medicine, Christian-Albrechts-University, Kiel, Germany; 3 Institut für Anästhesiologische Pathophysiologie und Verfahrensentwicklung, Universitätsklinikum Ulm, Ulm, Germany

## Abstract

In this commentary, the authors discuss two possible approaches in experimental studies. The first approach is to replicate an experimentation in order to confirm or not previously published results. The second one is more theoretical and consists in estimating the expected effect of all the components of the problem. When theoretical calculations suggest a theoretical failure that contradicts previous published results, investigators are between a rock and a hard place. Indeed, how can already published data and theoretical likelihood of failure be reconciled?

## Correspondence

The search for alternative routes to support oxygenation in case of severe lung failure is a very valuable concern in critical care medicine [1]. However, particularly in such a desperate clinical situation, an alternative route should significantly contribute to oxygenation without further jeopardizing the patient.

Old publications have suggested that the intestinal mucosa may act as a membrane allowing oxygen diffusion [2]. Thus, intestinal oxygen administration was proposed as a possible alternative route to improve respiratory failure-induced cyanosis in human. In another old study, oxygen was administered intravenously at rates up to 600–1200 mL/h without clear detrimental effects [3]. These publications are clearly provocative, and confirmation of the results is required.

In such situation, two scientific approaches, at first sight opposed, are possible.

The first approach, maybe the easier one, consists in reproducing the experiment in order to confirm or not the previously published results. This approach is largely used in biology and is the cornerstone of evidence-based medicine, allowing the assessment of the external validity of a study.

The second approach is to analyze, as far as possible, the theoretical components of the problem and to propose theoretical hypotheses. Secondarily, the theoretical hypotheses have to be confirmed by the experiment. This is typically the case in physics.

In this issue, Damiani et al. have used the first approach and conclude from their study that “in this rat model of hypoxemia, the intravenous infusion of gaseous O_2_ was unfeasible as it induced early mortality,” and that “although safe, both intravenous infusions of oxygenated Hartmann’s solution and bowel O_2_ administration were unable to improve arterial or tissue oxygenation.” In the setting of this particular experiment, when the authors made the detailed study plan, they could have theoretically estimated the oxygen amount delivered by the experimental methods (intravenous or intestinal route) using the well-known oxygen uptakes in rats and men, O_2_ solubility under normobaric conditions [4], and proper volume calculations. Thus, the authors could have predicted the failure of their confirmatory study; thanks to this estimation. Hence, the authors would have respected the 3Rs rule (Reduce, Refine, and Replace) recommended for the design of animal studies [5, 6]. For the present study, the theoretical approach may have been helpful in reducing (first R) the number of animals and would have predicted the failure of the experiment.

In the present study, the theoretical calculation was performed after the end of the experiment. In their discussion, the authors state that “retrospective theoretical calculations clearly showed that the experimental approach applied was unsuitable to produce any positive results.” What would the authors have done if they had the theoretical information before the experiment? This raises immediately an ethic concern, and the authors would have been between a rock and a hard place. Indeed, how can the already published data and the theoretical likelihood of failure be conciliated? Are we ready to accept in such situation only theoretical results to apply the Refine and Replace rules of the European directive on the protection of animals used for scientific purposes?

If not, the pragmatic approach is to perform the experiment in order to definitively answer the question and close the debate.
